# *In Vitro* Generated Hepatocyte-Like Cells: A Novel Tool in Regenerative Medicine and Drug Discovery 

**DOI:** 10.22074/cellj.2016.4362

**Published:** 2017-02-22

**Authors:** Kobra Zakikhan, Behshad Pournasr, Massoud Vosough, Marjan Nassiri-Asl

**Affiliations:** 1 Cellular and Molecular Research Center, Department of Molecular Medicine, School of Medicine, Qazvin University of Medical Sciences, Qazvin, Iran; 2 Department of Stem Cells and Developmental Biology, Cell Science Research Center, Royan Institute for Stem Cell Biology and Technology, ACECR, Tehran, Iran; 3 Department of Regenerative Biomedicine, Cell Science Research Center, Royan Institute for Stem Cell Biology and Technology, ACECR, Tehran, Iran; 4 Cellular and Molecular Research Center, Department of Pharmacology, School of Medicine, Qazvin University of Medical Sciences, Qazvin, Iran

**Keywords:** Hepatocyte, Cell Therapy, Gene Therapy, Drug Discovery

## Abstract

Hepatocyte-like cells (HLCs) are generated from either various human pluripotent stem cells (hPSCs) including induced pluripotent stem cells (iPSCs) and embryonic stem cells (ESCs), or direct cell conversion, mesenchymal stem cells as well as other stem cells like gestational tissues. They provide potential cell sources for biomedical applications. Liver transplantation is the gold standard treatment for the patients with end stage liver disease, but there are many obstacles limiting this process, like insufficient number of donated healthy livers. Meanwhile, the number of patients receiving a liver organ transplant for a better life is increasing. In this regard, HLCs may provide an adequate cell source to overcome these shortages. New molecular engineering approaches such as CRISPR/ Cas system applying in iPSCs technology provide the basic principles of gene correction for monogenic inherited metabolic liver diseases, as another application of HLCs. It has been shown that HLCs could replace primary human hepatocytes in drug discovery and hepatotoxicity tests. However, generation of fully functional HLCs is still a big challenge; several research groups have been trying to improve current differentiation protocols to achieve better HLCs according to morphology and function of cells. Large-scale generation of functional HLCs in bioreactors could make a new opportunity in producing enough hepatocytes for treating end-stage liver patients as well as other biomedical applications such as drug studies. In this review, regarding the biomedical value of HLCs, we focus on the current and efficient approaches for generating hepatocyte-like cells *in vitro* and discuss about their applications in regenerative medicine and drug discovery.

## Introduction

Nowadays human primary hepatocytes are regularly used, as the most important and efficient cells in the liver organ for biomedical applications, e.g. cell therapy and drug studies (1,3). Some evidences reported application of hepatocytes for cell therapy clinical trial of various liver disorders (4,5). Although limited access to sufficient human functional hepatocyte, due to the lack of availability of healthy donors as well as difficulties in hepatocyte long-term maintenance, are major problems in using these specialized cells (6). 

**Fig.1:**
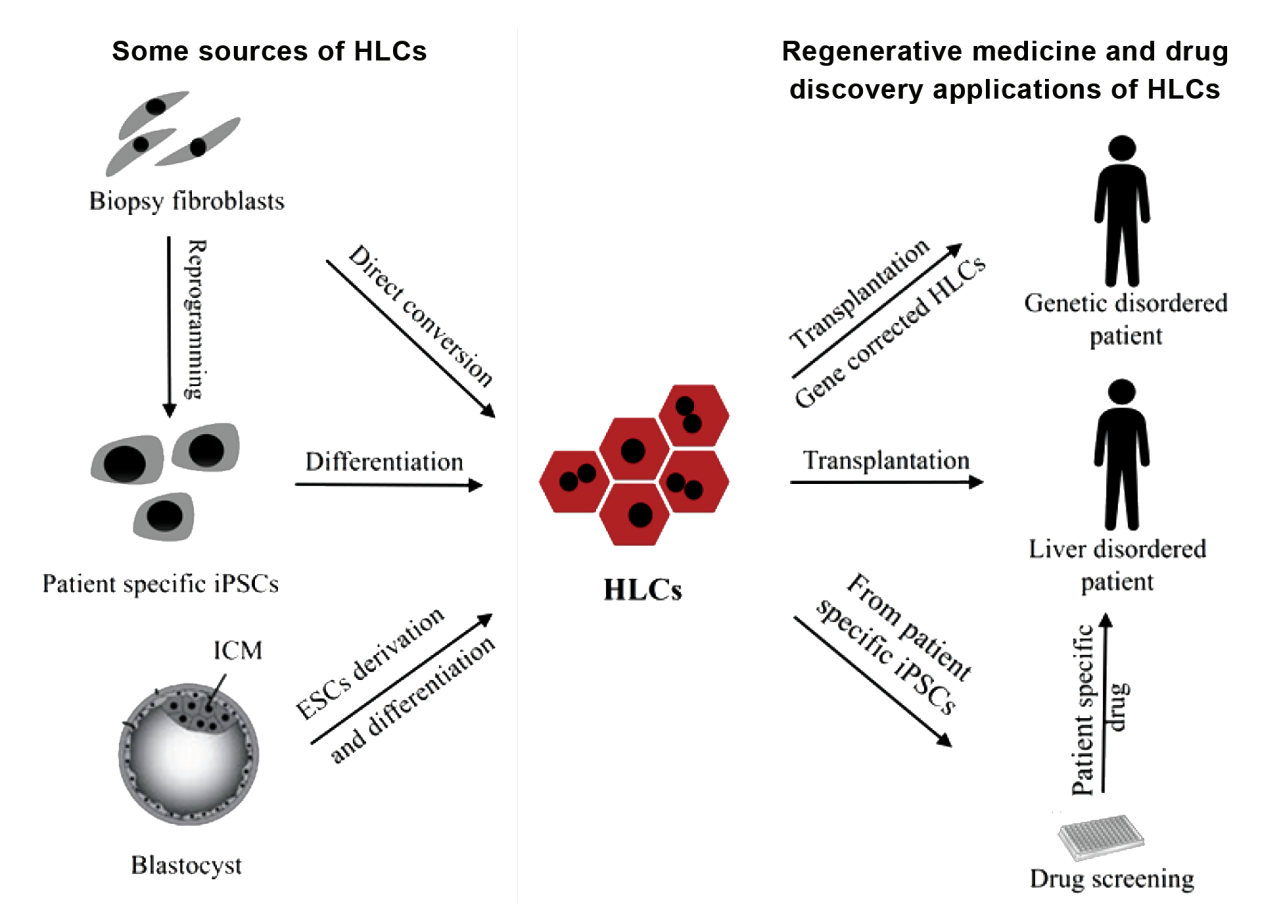
Main sources of HLCs and their applications in regenerative medicine and drug discovery. Diagram of some sources of HLC (Left): biopsy derived fibroblasts from liver disease patient can directly be converted into HLCs, by overexpression of liver specific transcription factors (TFs). Patient specific iPSCs generated by overexpression of Yamanaka factors (Oct4, Sox2, Klf4 and c-Myc) can also be differentiated to HLCs for further applications. Embryonic stem cells from ICM of blastocyst are other sources of HLCs. Diagram of some potential biomedical applications of HLC (Right): HLCs can be used for patients with end-stage liver disease. In addition, using iPSCs technology, monogenic disorders can be corrected in metabolic liver diseases at genome level and then healthy patient specific iPSC-derived HLCs could be a source for transplantation and decreasing signs of the disease. Drug screening after disease modeling, using patient specific iPSC-derived HLCs, to achieve new drugs for specific patients and individual drug administrations are another application of HLCs in the personalized medicine field. HLCs; Hepatocyte-like cells and iPSCs; Induced pluripotent stem cells.

Primary human hepatocytes, usually derived from the livers, are immunologically rejected for transplantation and therefore the yield and quality of the isolated hepatocytes are a limiting factor in any biomedical application (3, 7). Researchers has currently introduced another source of hepatocyte as possible substitute, known as hepatocyte-like cells (HLCs), which has been produced *in vitro*. HLCs are usually derived from human pluripotent stem cell (hPSCs), including human embryonic stem cells (hESCs) and induced pluripotent stem cells (iPSCs), gestational stem cells and mesenchymal stromal cells. Direct cell conversion is another method to generate HLCs (6, 8). Protocols to generate higher quality of HLCs are continuously improving and different research groups are working in this regard. Moreover, scaling up production of HLCs, using three-dimensional system (3D) in bioreactors, resulted in generating enough cells for any biomedical application (9, 10). In this review, we briefly described different methods to produce HLCs *in vitro* and explained some of their applications in research and regenerative medicine. Figure 1 presents regenerative medicine, drug study, some sources and applications of HLCs.

## Different types of produced hepatocyte-like cells in vitro

### Human embryonic stem cells-derived hepatocytese

ESCs, derived from the inner cell mass of blastocysts are immortalize cell type with ability to differentiate into all somatic cell lineages (11, 12). These primitive and highly undifferentiated cells were firstly isolated from mouse embryos (mESCs) (11) and the first hESCs line was successfully derived from *in vitro* fertilized human embryos (13). It has been shown that these cells with a high level of self-renewal ability and possibility to produce nearly all cell types, including "hepatocyte", can be used as an important tool for basic and clinical researches (14). There are two ways to produce HLCs through hESC: spontaneous differentiation and directed differentiation.

In the first approach, hESCs are aggregated to form human embryoid bodies (hEBs). These cell aggregates spontaneously start to differentiate into the three germ layers, including endodermal cells (15, 16). It has been shown that hESC can differentiate into hepatic-like cells through the EB formation, thus albumin-expressing cells have subsequently been detected in EBs (17, 18). Due to the low efficiency of spontaneous differentiation of hESCs, possibility of miscellaneous differentiation into any other cells and possibility of differentiation into non-homogeneous population of cells, scientists focused on the directed differentiation of hESCs into HLCs (14).

In this approach, several protocols have been developed to differentiate ES cells toward HLCs sequentially. In these protocols a series of growth factors and some other soluble factors which participate during liver development have been used in a stepwise manner, mimicking *in vivo* liver development (15, 19-22). Generally, these protocols have some specific steps. The first step is "Endoderm induction" whereby mainly activin is used. The second step is "Hepatic specification" using some factors like bone morphogenetic proteins (BMPs) and fibroblast growth factors (FGFs). "Hepatoblast (hepatic stem cell) expansion" and "Hepatic maturation" are respectively the other steps, developed by using specific growth factors like hepatocyte growth factor (HGF), epidermal growth factor (EGF), oncostatin M (OSM) and Dexamethasone (DEX) (8). The progress in each step is usually evaluated by specific markers. Bile duct cells are excluded based on their specific markers in order to have a more homogenous population of hepatocytes (23). Siller et al. (24) recently presented a growthfactor- free protocol using only small molecules to induce HLC differentiation to pluripotent stem cells. CHIR99021, as potent pharmacological glycogen synthase kinase-3 (GSK-3)-specific inhibitor activating wingless-type MMTV integration site family (Wnt) signaling pathway during hepatocyte differentiation (25), was used to induce definitive endoderm formation, followed by treatment with dimethyl sulfoxide (DMSO), dexamethasone, hydrocortisone-21-hemisuccinate and Ile-(6) aminohexanoic amide (dihexa), as a small molecule that is an agonist of HGF (26), to drive hepatic maturation. The polygonal HLCs were generated, which expressed hepatic speciﬁc markers like albumin, AFP and alpha-1 antitrypsin (A1AT) with proper functionalities. In addition, some other groups have tried to improve current hepatic differentiation protocols with different strategies like using other appropriate cells for co-culture, monolayer culture or using 3D cell aggregates, through differentiation protocols (9, 27-31).

### Induced pluripotent stem cells-derived hepatocytes

HLCs can be efficiently produced by iPSCs. iPSCs were introduced by the forced expression of a set of transcription factors (*Oct4, Sox2, Klf4,* and *c-Myc* genes) using a retroviral vector in somatic cells. These pluripotent reprogrammed cells were called iPSCs. Like ESC lines, iPSCs can differentiate into all three cell lineages including endoderm, while they have intensive proliferation *in vitro* (32, 33). In other studies, researches were focused on alternative ways to generate iPSC lines, different from integrative viral-mediated strategies, e.g. using excisable viral vectors (34), RNA-Sendai virus vectors (35), episomal plasmids transfections (36), miRNA (37) or mRNA transfections (38) as well as using only chemical compounds (39).

There are many studies which show that iPSCs can differentiate into HLCs (24, 40-43). These generated HLCs had some characteristics of human hepatocytes, particularly in morphology and phenotype (44), but regarding functional assays and metabolic activity, these cells were similar to immature hepatocytes (44, 45). Therefore, numbers of research group have been trying to increase the efficiency of differentiation of iPSCs to HLCs in 2D and 3D with different differentiation protocols (9, 24, 40, 46, 47). In both 2D and 3D differentiation protocols, the extracellular matrix, additional cell-cell interactions, the media and supplements, i.e. growth factors and cytokines, ensure the successful differentiation of iPSCs to HLCs (48). Recently, it has been shown that in 3D culture, the maturity of HLCs derived from iPSCs was increased (46). Moreover, spatially patterning of the cells, known as self- organization, to give rise to "organoid structures" has been introduced in 3D cultures (49). These structures can be expanded without limiting, cryopreserved as biobanks and easily manipulated using techniques established for 2D culture (50). Takebe et al. (29) in 2013 demonstrated when human iPSCs were co-cultured with endothelial and mesenchymal cells, they were self-organized *in vitro* into structures like small liver organoids, also called "liver buds", while they could be transplanted. Moreover, in other studies, researchers have tried to manipulate current protocols for more hepatocyte maturity, including drug metabolism activity (51).

### Gestational stem cell-derived hepatocytes

Another source of HLCs is gestational stem cells derived from umbilical cord, umbilical cord blood, placenta, and amniotic fluid. This type of stem cells, which is easily accessible, can generate other cell lineages *in vitro* and *in vivo* (52, 53). Studies showed that human umbilical cord stem cells could differentiate into HLCs *in vitro*, with hepatocyte-like morphology and high-level expression of hepatic lineage markers. These cells could differentiate into HLCs *in vivo* either after injection into the NOD-SCID mice with induced liver damage (54, 55). In addition, human placenta-derived multipotent cells can also differentiate into other cell types including HLCs with primary hepatocyte characteristics (56). Gestational stem cells do not form teratomas or teratocarcinomas in humans, while they have a high proliferation rate and differentiation potential. Because of the plasticity and accessibility of these stem cells, many cord blood banks have been established for the collection and storage of these cells for future applications (57).

### Mesenchymal stromal cells derived hepatocytes

Generation of HLCs from mesenchymal stromal cells (MSCs) using different sources such as bone marrow (BM-MSCs), umbilical cord blood (UC-MSCs), stem cell-derived (ESC-MSCs) and adipose tissue MSCs (Ad-MSCs) have been previously described (58). These types of stem cells are fibroblast-like, plastic-adherent and multipotent cells, rapidly expanding *in vitro* under standard conditions. MSCs have low immunogenicity and possess immunomodulatory properties, so they are commonly used in cirrhosis (59-63). They can differentiate into HLCs, expressing particular hepatic genes and presenting some metabolic activities (64). Culturing BM-MSCs in hepatocyte-conditioned medium without any growth factors can induce hepatic cell differentiation (65). Combination of HGF, nicotinamide and dexamethasone in MSCs culture medium could induce hepatic fate in MSCs (66). Moreover, other growth factors like insulin-like growth factor-I in combination with liver specifi c factors have been reported to differentiate MSCs into HLCs (67).

### Direct cell conversion to hepatocyte-like cells

Direct conversion of adult cells like fibroblasts to other mature or progenitor somatic cells is an alternative way to bypass the pluripotent iPSC step, mainly by ectopic expression of particular cell-specific transcription factors (TFs). In this regard, functional cells are directly generated, which are useful in advanced clinical applications as well as basic science studies (68, 69). By this new method, known as "transdifferentiation", different research groups successfully converted mouse and human fibroblasts to other lineages, including neurons, cardiomyocytes and hepatocytes, the latter one was also called induced hepatocytes (iHeps). Sekiya and Suzuki (70) showed that a combination of *Hnf4a* with *Foxa1, Foxa2* or *Foxa3* could convert mouse adult fibroblasts into iHeps and this generated cells could repopulate and save the genetically modified mice model, with deficiency in the fumarylacetoacetate hydrolase (Fah) activity, leading to the accumulation of metabolites of tyrosine that are toxic to native hepatocytes.

In another study, ﬁbroblasts were converted to iHeps by the transduction of *Hnf1a plus Gata4* and *Foxa3*, and inactivation of p19Arf. This iHeps could repopulate the livers of Fah- /- mice, increasing the survival rate in the recipients (71). Similar technique was used to generate human induced hepatocyte (hiHeps) by the forced expression of *HNF1α, HNF4α,* and *HNF6* with *ATF5, PROX1, C/EBPβ* as the maturation factors and P53-shRNA (72). In another study by increasing the expression of *FOXA3, HNF1α, HNF4α* and *FOXA2*, iHeps were generated (73). In both studies, *in vitro* gene expression proﬁles of hiHeps were similar to mature human hepatocytes and they showed *in vivo* functionality in FRG (Fah-/-/Rag2- /-/Il2rg) mouse model. In another strategy, Yamanaka factors were used for generation of an epigenetic instability, along with the small molecules and/or a cocktail of *Hnf4a, Cebpa* and *Nr1i2* under hepatic inducing conditions (74). In addition, it has been shown that Kdm2b as an epigenetic modulator with HNF4α and Foxa3 could accelerate generation of iHeps in hepatic media (75).

## Applications of in vitro produced hepatocyte-like cells

### Applications of hepatocyte-like cells in cell therapy of acquired liver diseases

There are many patients suffering from liver disease worldwide. Many of these patients with acquired liver diseases, such as acute liver failures (ALF), fulminant hepatic failure and chronic liver diseases can benefit from cell therapy, specially hepatocyte transplantation (2, 76). In acute liver failure and fulminant hepatic failure, liver metabolic functions are seriously deteriorated following the loss of hepatocytes mass caused by toxins, drugs and hepatotrophic viruses. In chronic liver diseases, severe alteration of hepatic microarchitecture is followed by generation of fibrotic areas (77). Recently ﬁbrosis regression was reported by in vivo hepatocyte transplantation. In these studies myofibroblasts, recruited in fibrotic areas, were reprogrammed into hepatocytes (78, 79).

Up to now many clinical trials with hepatocyte transplantation have been successfully performed with cost effeciecy and simply doing cell administration by intravascular injection rather than surgery (1, 4, 80-84). Moreover, it is possible to use cryopreserved hepatocytes and may even be transplanted them repeatedly (85). Due to the limitations for cell therapy with hepatocyte, such as insufficient numbers and low viability of cells, *in vitro* generated HLCs offer a new arena for basic studies and a potential source for possible use in therapy in the future (6, 14).

Human iPSCs derived hepatocyte-like cells (iPSCs-HLCs) presented a new platform for the liver cell therapy, but there is no registered clinical trial using these cells for cell therapy (86). However, in a mouse model it has been shown that iPSCs-HLCs could efficiently be engraftd into the liver with normal function (40). These cells were applied in lethal fulminant hepatic failure in non-obese diabetic severe combined immunodeficient mice and rescued them after cell therapy (87). Because of the ethical advantages of iPSCs and using autologous starting cells, as an important step toward "personalized medicine", it seems that iPSCs-HLCs have potential of clinical applications in future of liver diseases (6, 88). Recently, iPSCs have been generated without viral vectors and transgene-free sequences by non-integrating episomal vectors (36). Transdifferentiation of MSCs into HLCs has already been reported and these generated HLCs has been characterized *in vitro* and in vivo (58). Animal model studies showed that in vitro pre-differentiated MSCs could boost liver repopulation and functionality of hepatic cells (58, 89). Moreover, co-transplantation of iPSCs-HLCs and MSCs could be a suitable option for the treatment of end-stage liver disease, due to the paracrine effects of drived MSC trophic factors (76). In Table 1, current cell sources for liver cell therapy, as well as some HLCs from various sources, as potential cell types appropriate for cell therapy, are described.

**Table 1:** Cell therapy of various liver disease with potential pluripotent stem cells-derived HLCs and other appropriate cells


Cell sources	Role in disease types	Clinical trial	Disadvantage	Reference

Hepatocytes	Metabolic liver disorderLiver disorders in infantsAutoimmune liver disorders	Yes	Possibility of infection with hepatitis virusesDecreased engraftmentability in injured liverLimited access	(90)
hESCs-HLCs	Liver disordersMetabolic liver disorder	No	Unknown maintenance in long-term	(91)
hiPSCs-HLCs	Liver disordersMetabolic liver disorder	No	No fully function Unknown maintenance in long-term	(92)
MSCs	Liver disordersCirrhosis	There are numbers of clinical reports	Some negative results in clinical studies	(93)


HLC; Hepatocyte-like cells, hESCs; Human embryonic stem cells, hiPSCs; Human induced pluripotent stem cells, and MSCs; Mesenchymal stem cells.

## Challenges in cell therapy with hepatocyte-like cells

The ESCs/ iPSCs derived HLCs with current protocols have a fetal-like phenotype, rather than a mature hepatocyte phenotype, however, it is possible to induce more maturation *in vivo* (45). Heterogeneous populations of HLCs, including differentiated and undifferentiated cells may increase the risk of tumorogenicity due to the high proliferation capacity of undifferentiated cells (94, 95). Then enrichment strategies create a new platform for generating HLCs for future clinical and pharmaceutical application (9). Furthermore, for any potential clinical use of HLCs derived from pluripotent cells, the iPSCs and ESCs should be generated in good manufacturing practice (GMP) condition as an appropriate system for protection of products and control them according to accridated quality standards. Therefore, many research groups are working to improve the protocol for generating HLCs from these pluripotent cells. Up to now, some studies showed that transplantation of ESC-derived HLCs in animal models could improve hepatic function (19, 96-101), but ethical concerns and regulatory issues, immunologic rejection and tumorogenicity are still the main limiting factors. To avoid time consuming procedure in the generation of HLCs from pluripotent stem cells under monolayer culture, we need to produce HLCs in 3D suspension culture for the clinical applications (102). Vosough et al. (9) described a scalable stirred-suspension bioreactor culture of functional HLCs from the hPSCs. After intrasplenic transplantation of these HLCs in acute liver injury, an increased survival rate and efficient engraftment of functional cells were observed.

## Applications of hepatocyte-like cells in cell therapy of acquired liver diseases

iPSC technology provides the possibility of "gene correction" on patient somatic cells. The gene correction on iPSCs can be applied for patients with monogenic inherited metabolic liver diseases, like Alpha-1 antitrypsin deficiency and Wilson’s disease (mutation in *ATP7B* gene). After *ex vivo* gene correction, iPSCs can differentiate into hepatocyte and then be transplanted to the patient (76). In gene correction of Alpha-1 antitrypsin deficiency in iPSCs, scientists showed that a combination of two targeted gene technologies, zinc finger nucleases (ZFNs) and PiggyBac (PB) technology, which were significantly efficient gene-targeting technology, could correct a point mutation (Glu342Lys) and then corrected iPSCs-HLCs could restore the structure and function *in vitro* and *in vivo* (103). In another study, researchers tried to generate iPSCs from a Chinese patient with Wilson’s disease (WD) that bears the R778L Chinese hotspot mutation in the ATPase Cu^2+^ transporting beta polypeptide (*ATP7B*) gene.

After gene correction using a self-inactivating lentiviral vector that expresses codon-optimized *ATP7B*, these iPSCs were differentiated into HLCs with copper metabolism capacity, which reversed the functional defect *in vitro*. These studies could introduce a new way for generating disease modeling valuable for screening alleviate compounds or gene therapy approaches (104).

Recently, some new technologies such as clustered regularly interspaced short palindromic repeats (CRISPR)/Cas based RNA-guided DNA endonucleases , as a new and powerful genome editing tool, allow precise gene editing in liverbased monogenic disorders in animal models by either permanently deleting/inserting specific genetic sequences or adding/removing epigenetic information temporarily with minimal off-target modifications (105, 106). In a new study, researchers used dual adeno-associated virus (AAV) vectors to deliver the CRISPR/Cas9 components, one expressing Cas9 and the other expressing a guide RNA as well as the donor DNA, to newborn mice with a partial deficiency in the urea cycle disorder enzyme, ornithine transcarbamylase (OTC), which resulted in improvment of their survival rate. They limited any off-target activity by using a liver-specific promoter for Cas9, ensuring its expression only within liver cells (107). In another study, viral and non-viral delivery systems were used. So, gene editing was accomplished with a combination of lipid nanoparticle–mediated delivery of Cas9 mRNA with adeno-associated viruses encoding a sgRNA and a repair template, to induce repair of a human hereditary tyrosinemia disease gene in adult mouse models. In this report, disease symptoms, such as weight loss and liver damage, were rescued. In addition, the efficiency of correction was reported less than 6% of hepatocytes after a single application (108). These presented results and efficacy of corrections were suggested potential utility of CRISPR-mediated gene repair for genetic diseases.

## Applications of hepatocyte-like cells in cell therapy of acquired liver diseases

HLCs, obtained from various sources, have cytochrome P450 (CYPs) activity, which is crucial for metabolism of xenobiotics and drugs (Table 2) (45, 73, 109). Although one of the main goal of improving differentiation protocols in different studies is increasing the functionality of generating cells in drug metabolism and appropriate CYPs activity. Recent studies tried to show an increasing activity of HLCs in drug metabolism (51, 72, 109). To measure the capability of HLCs for drug metabolism, particular substances were introduced, including phenacetin (CYP1A), bupropion (CYP2B6), diclofenac (CYP2C9) and midazolam (CYP3A) (109). Before evaluating the HLCs, they should be treated with an appropriate inducer, like phenobarbital and rifampicin. In addition, mRNA and protein expression of important CYPs in the presence and absence of inducers can be checked in HLCs (70, 109-112).

If researchers can generate HLCs with the ability of drug metabolism, these cells might be replaced with primary hepatocyte as a "gold standard" for drug metabolism and drug toxicity tests (86). Activity and expression of drug transporters are another characteristic that sometimes were assessed in generating HLCs in various studies to evaluate the quality of them compared to primary hepatocytes. Therefore the activity of the uptake transporters such as organic anion transporting polypeptides (OATPs), OATP1B1 and Na^(+)^-taurocholate cotransporting polypeptide (NTCP) as well as the efﬂux transporter bile salt export pump (BSEP) were evaluated (109).

Another important application of HLCs, especially with iPSC technology, is in discovery of new and safe drugs, small molecules and components to alleviate the respective property after high-throughput screening. In this way, HLCs produced by patient specific iPSCs were checked by the array of drugs or components to find new drugs or investigating toxicity of drugs. Moreover, testing drugs for iPSC that belong to an individual, admirably help to choose the best drug among various candidates and also determine the best dose of the chosen drug for every patient (14, 86, 113). Undoubtedly, this is the best way for "personalized drug administration" which will guide toward the future medicine. In addition, recently bioengineering tools such as microfluidicbased cell culture device opened new window to drug studies with HLCs (114). This controlled system allows exact spatial and frequent delivery of media, drugs and signaling factors to live cells (115). Giobbe et al. (116) reported a microfluidic system on the chip to differentiate PSCs into HLCs to predict drug toxicity. Further more, by providing a controlled microenvironment for generating HLCs, microfluidic systems make it feasible to study the candidate or new drug metabolism and drug-response screening in high-throughput testing (117, 118).

Recent developments in humanized liver models present a promising horizon in the future preclinical applications of generated hepatocytes *in vivo*, especially for drug discovery (3). It has been reported that human hepatocyte can be transplanted into the animal models and they can be repopulated in the host liver (119). Highly immunodeficient FRG [Fah(/) Rag2(/) Il2rg (/)] mice are currently the best model for repopulation of human hepatocyte in animals. This model is T, B and NK cells deficient, in addition to deficiency in the fumarylacetoacetate hydrolase (Fah) activity, as an enzyme catalyzing the last step of tyrosine metabolism (120). Researchers reported that FRG mice liver can be repopulated efficiently with human hepatocyte and display a serum lipoprotein profile similar to human apolipoproteins.

**Table 2:** Examples of recently reported CYP enzymes activity and drug metabolisms in generated HLCs from different sources


Cell sources	CYP enzymes/Drug metabolism	Method of analysis	Inducer	Reference

hESC	CYP1A1, CYP1A2, CYP3A4, CYP7A1, CYP1B1, CYP2B6, CYP2C9,CYP2C19, CYP2D6, CYP2E1	PCR	No	(121)
hESC, hiPSC	CYP1A2, CYP3A4, CYP3A7, CYP2D6, CYP2C9, CYP2C19	Immunohistochemistry/ luminescence based kit	Phenobarbital, rifampicin and acetaminophen	(122)
iPSCs	CYP3A4	luminescence based kit	No	(46)
iPSCs	CYP1A1	EROD	Ethoxyresorufin, dicumarol	(123)
Fibroblast (direct conversion)	CYP1A2, CYP2B6, CYP2C9, CYP2C19, CYP3A4, CYP2C9, CYP2C19, CYP1A2, CYP3A4Testosterone, midazolam, phenacetin, bupropion, diclofenac, S-mephenytoin	qPCR, IF,HPLC-MS	Rifampicin, b-naphthoﬂavone, phenobarbital	(72)
Fibroblast (direct conversion)	CYP1A2, CYP2A6, CYP2B6, CYP2C8, CYP2C9, CYP3A4/phenacetin, coumarin, dextromethorphan	qPCR,LC-MS/MS	3-methylcholanthrene,phenobarbital, or rifampicin	(73)
AT-MSC	CYP1A1, CYP1A2, CYP2A1, CYP2C7, CYP2C12, CYP2E1, CYP3A1Phenacetin, coumarin, chlorzoxazone	qPCR,LC-MS/MS	No inducer,3-methylcholanthrene, phenobarbital,and acetone	(124)
Human umbilical cord-derived MSC	CYP3A4	Liquid chromatography	Midazolam	(125)


HLCs; Hepatocyte-like cells, hESC; Human emberyonic stem cell, iPSCs; Induced pluripotent stem cells, AT-MSC; Adipose-derived mesenchymal stem cells, qPCR; Quantitative polymerase chain reaction, IF; Immunofluorescence, HPLC-MS; Liquid chromatography-mass spectrometry, and LC-MS/MS; Liquid chromatography tandem-mass spectrometry.

They displayed that these humanized FRG mice have capacity to be a suitable model for atherosclerosis and cholesterol metabolism. Moreover and interestingly, in these mice drug metabolizing enzyme system were humanized either (126). Hickey et al. (127) represented a new Fah(/) pigs, as a large animal model. Fah deficiency is an utero lethal difficulty in pigs that is correctable with administration of 2-(2-nitro- 4-trifluoromethylbenzoyl)-1,3 cyclohexanedione (NTBC). After withdrawing NTBC, FAH−/− pigs died due to acute liver failure. This animal model may be suitable platform to generate HLCs with further functionality. Another research group tried to improve repopulation efficacy of humanized liver in chimeric mice with transduction of HLCs by adenovirus vector (Ad-FNK) to express FNK. Overexpression of FNK resulted in apoptosis inhibition in HLCs. In this study, Ad-FNKtransduced human iPSC-HLCs transplanted into urokinase-type plasminogen activator-transgenic severe combined immunodeficiency (uPA/SCID) mice and assessed the effectiveness of repopulation. Human albumin levels, human hepatocyte-related genes and proteins in the transplanted mice were significantly increased in this model (128). Briefly, human pluripotent stem cell-derived HLCs are appropriate and promising sources for the generation of humanized liver models.

Achieving adequate and appropriate HLCs, is an important route for cell therapy of patients with end-stage liver failure (48). There are some potential sources that researchers have focused on it in recent years for obtaining functional HLCs, including mainly stem cells-based sources like MSCs, pluripotent stem cells, hESC and induced pluripotent stem cell (iPSC) lines, as well as direct lineage conversion of adult somatic cells to HLCs as a new strategy (129). HLCs exhibit many phenotypes and some functional traits of mature hepatocytes (6). Up to now there is no registered clinical trial using HLCs, but there are some considerable potential advantages of HLCs against primary hepatocytes, especially for iPSC-HLCs, including potential of large scale production, patient-specificity of iPSC-HLCs preventing transplanted cell immunorejection, possibility of gene editing of autologous iPSCHLCs with non-integrated tools to treat inherited genetic liver diseases prior to differentiation and transplantation, especially with helping new tools such as the most widely used engineered CRISPR/ Cas system (77, 107, 108). The CRISPR/Cas9 method, as a powerful genome editing system, successfully corrected point mutations in A1AT deficiency disease-specific iPSCs (105). Moreover, *PCSK9* gene was mutated by CRISPR/Cas9 system in mouse liver for changing the lipid profile of animals, i.e. decreasing plasma cholesterol level and increasing LDL receptors (130). In another study, an adenovirus based CRISPR/Cas9 system for *in vivo* gene editing precisely knocked-out the *CEBPα* gene, as an important transcription factor for metabolic genes in the liver organ (131).

Various studies have conﬁrmed the feasibility of generating and cultivating human pluripotent stem cells in stirred suspension bioreactors (9, 132). Using this technology helped us to move forward to practical applications, requiring a large number of cells for treatment and high- throughput drug screening (48).

HLCs have been shown to be a powerful *in vitro* system not only to study patient-specific disease model and some human liver disease, i.e. viral hepatitis and plasmodium infection (133- 136), but also to drug study, especially with the help of modern techniques such as microfluidicbased cell culture platforms. This system either allows to improve HLCs generation by providing monitoring of culture parameters or helping to co-culture of HLCs with the other cells, even from other organs, make it possible to investigate unintended systemic side-effects of therapeutic agents and their metabolites (137). Moreover, human pluripotent stem cells-derived HLCs provide infinite and genetically defined sources for humanized liver models. These animal models will provide many *in vitro* applications, including highthroughput drug screening, toxicology and further applications in liver assist devices (138).

Finding potential sources and strategies for generating HLCs open a new window in liver regenerative medicine, but many considerable experimental challenges remain to be solved including finding new methods or biomolecules improving the efficiency as well as extending differentiation of these cells and metabolic activity of generated HLCs. Besides, finding new improved methods to enrich, purify and large-scale production of HLCs are necessary. On the other hand, development of suitable animal models and efficient HLCs delivery strategies should be considered for improvement of future clinical use (139).

## Conclusion

*In vitro* generation of HLCs using pluripotent or non-pluripotent cells via differentiation and direct conversion to hepatocytes provides potential applications in regenerative medicine, via cell and gene therapies for liver diseases and drug discovery. At this time, considerable experiments continue to increase the functionality of generated HLCs to introduce them as a suitable replacement for primary hepatocytes.
